# Editorial: Tissue Stem Cells During Trauma: From Basic Biology to Translational Medicine

**DOI:** 10.3389/fcell.2022.914582

**Published:** 2022-05-26

**Authors:** Guohui Liu, Guozhi Xiao, Jiacan Su, Ren Xu, Zhidao Xia

**Affiliations:** ^1^ Department of Orthopedics, Union Hospital, Tongji Medical College, Huazhong University of Science and Technology, Wuhan, China; ^2^ Guangdong Provincial Key Laboratory of Cell Microenvironment and Disease Research, Shenzhen Key Laboratory of Cell Microenvironment, Department of Biochemistry, School of Medicine, Southern University of Science and Technology, Shenzhen, China; ^3^ Department of Orthopedics Trauma, Shanghai Changhai Hospital, Second Military Medical University, Shanghai, China; ^4^ State Key Laboratory of Cellular Stress Biology, School of Medicine, Xiamen University, Xiamen, China; ^5^ Institute of Life Science, College of Medicine, Swansea University, Swansea, United Kingdom

**Keywords:** stem cells, translational medicine, osteoporosis, drugs, biophysical stimuli

Bone is a metabolically active organ that undergoes regular and ongoing remodeling throughout the lifespan, which is crucial for the maintenance of mineral metabolism and normal skeletal structure (Salhotra et al., 2020). The dynamic balance between bone formation and bone resorption is essential to maintain normal bone homeostasis. It is generally accepted that osteoporosis is attributed to an imbalance between bone formation and resorption during bone reconstruction under certain conditions, such as senescence, estrogen deficiency and use of glucocorticoid, whereby the rate of bone absorption is greater than that of bone formation, resulting in accelerated bone turnover and rapid bone loss.

Osteogenic differentiation is the key process in bone formation, and the decreased osteogenic differentiation ability of mesenchymal stem cells (MSCs) leads to decreased bone formation, which has been proven to be responsible for multiple bone disorders, including osteonecrosis of the femoral head and osteoporosis. Multiple clinical trials and animal experiments have demonstrated the beneficial effects of MSCs in preventing bone loss (Wang et al., 2021). Thus, MSCs may provide a therapeutic approach for patients suffering from osteoporosis, potentially contributing to promoting osteogenic differentiation, enhancing bone formation, and influencing the progression of bone remodeling (Jiang et al., 2021). However, current available therapies have some side effects, including muscle cramps, increased serum or urine calcium, etc, which may lead to decreased treatment compliance. Therefore, the continuous search for novel, effective drugs with low side effects has become a commitment to humanity.

Lin et al. found that polydatin (POL) may have a promising therapeutic effect in osteoporosis by bioinformatic analyses, and POL treatment exhibited an increase in the ALP-positive area, the alizarin red-positive area and levels of osteogenic gens, including collagen type I alpha 1 (COL1A1), alkaline phospatase (ALP), osteocalcin (OCN) and Runx2, through MAPK signaling pathway *in vitro* Lin et al., 2021 Another studies supplemented that POL could improve osteogenic differentiation of MSCs potentially via BMP2-Wnt/β-catenin signaling pathway (Shen et al., 2020), Nrf2 signaling (Chen et al., 2016) and regulating osteoprotegerin (OPG) and RANKL levels *in vivo* and *in vitro* (Zhou et al., 2016). These results demonstrated that POL treatment could increase bone mass *via* activation of multiple signaling molecules, but the side effects and the strength of the effect are unknown compared the current available drugs. This can only be clarified by a large randomized trial.

Hesperidin, a flavanone glycoside highly abundant in citrus fruits, has exhibited a protective role in osteoporosis through enhancing osteogenic differentiation and inhibition of osteoclastogenesis (Liu et al., 2019; Miguez et al., 2021; Zhang et al., 2021), but little is known about the role of hesperidin in the dexamethasone-induced osteoporosis. Zhang et al. indicated that hesperidin showed enhanced osteogenic differentiation and partially reversed dexamethasone-induced inhibition of osteogenic differentiation by p53 signaling pathway *in vitro*, suggesting the compound may be a promising candidate against dexamethasone-induced osteoporosis (Zhang et al., 2021). However, hesperidin in combination with calcium supplementation appears to have better effect in preserving bone in postmenopausal women compared with hesperidin (Martin et al., 2016). Given that hesperidin was widely found in vegetables and fruits, the study by Zhang et al. may provide evidence to support that food therapy could treatment or prevent dexamethasone-induced osteoporosis to some extent, but the effective dose of the compound is needed to be identify.

Xie et al. demonstrated that SHIP1 activator AQX-1125 not only simulated differentiation of bone mesenchymal stem cells (BMSCs) into osteoblasts and osteoblast matrix mineralization, but inhibited osteoclast formation and function in a dose-dependent manner, and subsequently reversed estrogen deficiency-induced bone loss. AQX-1125 is first-in-class, oral SHIP1 activator in clinic, and a clinical trial regarding the effect of the SHIP1 activator AQX-1125 reported that it was well tolerated and low side effect (Nickel et al., 2016). Therefore, AQX-1125, maybe have some unique advantages compared with current available therapies, such as few side effects, easily accessibility and dual effects between osteogenesis and osteoclastogensis. The short-term efficacy of AQX-1125 may be excellent in rescuing bone loss, but the long-term administration may lead to low bone turnover, resulting in high bone strength but increased bone fragility.

A combination of biophysical stimuli and drugs therapy may be a future direction in treatment osteoporosis. Given the inefficiency of current biomaterials and bioactive molecules, biophysical stimulation for MSCs osteogenesis, including internal structural stimulation, external mechanical stimulation and electromagnetic stimulation, showed promising potential to prevent and treat osteoporosis, although specific osteoinductive mechanisms remains unclear (Hao et al.; Zhang et al., 2021). Furthermore, application of biophysical stimuli might augment the therapeutic benefit and reduce drugs dose, subsequently reducing the side effects. Therefore, it appears to be an ideal adjunct on top of antiosteoporosis drugs.

In summary, the Research Topic indicated that the efficacy of these therapies and its mechanism against osteoporosis, highlighting the fact that strategies for enhanced osteogenic differentiation of MSCs in osteoporosis are effective. As researchers come round to the understanding that bone is a mechanosensitive tissue, biophysical stimulations offer some unique advantages for enhancing osteogenic differentiation of MSCs. Furthermore, drugs therapy in combination with biophysical stimuli may be as a first line therapy in the future. We predict that this Research Topic will continue to rapidly evolve and attract more researches to develop more antiosteoporosis therapies with few side effects.
